# Systematic Review, Quality Assessment, and Synthesis of Guidelines for Emergency Department Care of Transgender and Gender-diverse People: Recommendations for Immediate Action to Improve Care

**DOI:** 10.5811/westjem.60632

**Published:** 2023-12-20

**Authors:** Michael I. Kruse, Alexandra Clarizio, Sawyer Karabelas-Pittman, Blair L. Bigham, Suneel Upadhye

**Affiliations:** * McMaster University, Department of Family Medicine, Hamilton, Ontario, Canada; † McMaster University, Michael G. DeGroote School of Medicine, Hamilton, Ontario, Canada; ‡ Queen’s University School of Medicine, Kingston, Ontario, Canada; § Scarbrough Health Network, Department of Critical Care, Toronto, Ontario, Canada; ¶ McMaster University, Division of Emergency Medicine, Hamilton, Ontario, Canada

## Abstract

**Introduction:**

We conducted this systematic review to identify emergency department (ED) relevant recommendations in current guidelines for care of transgender and gender-diverse (TGD) people internationally.

**Methods:**

Using PRISMA criteria, we did a systematic search of Ovid Medline, EMBASE, and CINAHL and a hand search of gray literature for clinical practice guidelines (CPG) or best practice statements (BPS) published until June 31, 2021. Articles were included if they were in English, included medical or paramedical care of TGD populations of any age, in any setting, region or nation, and were national or international in scope.

Exclusion criteria included primary research studies, review articles, narrative reviews or otherwise non-CPG or BPS, editorials, or letters to the editor, articles of regional or individual hospital scope, non-medical articles, articles not in English, or if a more recent version of the guideline existed. Recommendations relevant to ED care were identified, recorded, and assessed for quality using the AGREE-II and AGREE-REX criteria. We performed interclass correlation coefficient for interrater reliability. Recommendations were coded for the relevant point of care while in the ED (triage, registration, rooming, investigations, etc.).

**Results:**

We screened 1,658 unique articles, and 1,555 were excluded. Of the remaining 103 articles included, seven had recommendations relevant to care in the ED, comprising a total of 10 recommendations. Four guidelines and eight recommendations were of high quality. They included recommendations for testing, prevention, referral, and provision of post-exposure prophylaxis for HIV, and culturally competent care of TGD people.

**Conclusions:**

This is the most comprehensive review to date of guidelines and best practices statements offering recommendations for care of ED TGD patients, and several are immediately actionable. There are also many opportunities to build community-led research programs to synthesize and inform a comprehensive dedicated guideline for care of TGD people in emergency settings.

## INTRODUCTION

Transgender and gender-diverse (TGD) patients comprise 0.3–0.6% of the North American population and may represent up to 1.2–4.1% of the adolescent population.^1^ ^–^ ^3^ Care of this population presents unique challenges in many practice settings, including emergency departments (ED).^4^ While ED avoidance has been high among TGD people due to systemic discrimination,^5^ ^,^ ^6^ ED use has also been found to be higher because of a lack of access to TGD-competent health services in primary and specialist care.^7^ As a result of these barriers and compounded by minority stress,^8^ ^–^ ^10^ The TGD populations experience a higher disease burden throughout their lifespan, including much higher rates of mental illness, self-harm, and substance use disorders.^11^ ^–^ ^13^ This has the potential to result in TGD people presenting with more severe illness when they come to the ED and requires an approach that does not recapitulate barriers they have experienced in the past to facilitate better care.

Clinical practice guidelines (CPG) have been defined by the Institute of Medicine as “…statements that include recommendations intended to optimize patient care that are informed by a systematic review of evidence and an assessment of the benefits and harms of alternative care options.”^14^ Best practice statements (BPS) are less evidence-driven and can include a consensus statement or practice advisory from an expert group, or a position statement or position paper from professional societies.^15^ Both can present standardized approaches to evidence-informed clinical care and are often adapted to meet local needs in the form of clinical manuals targeted at front-line clinicians and healthcare workers. There have been several reviews of CPGs for the care of TGD people in the recent past.^16^ ^–^ ^18^ Not all guideline recommendations can be successfully adopted/adapted into different clinical working environments, and there are none that focus on care in the ED. There are publications from the Emergency Medicine Residents’ Association and the American College of Emergency Physicians that speak directly to care of TGD populations in the ED but they do not represent the more rigorous systematic process of a CPG. The former is a clinical training manual, and the latter was published after search for this current study was completed.^19^ ^,^ ^20^

Previous work has demonstrated a paucity of research relevant to ED care of TGD patients.^22^ Our overall goal in conducting this systematic review was to identify and evaluate current practice recommendations that inform the care of TGD populations in ED settings.

## METHODS

This was a PRISMA-based systematic review of guideline recommendations, followed by application of the AGREE II and REX assessment tools for recommendation quality and applicability (available at www.agreetrust.org). The trial was registered at the Open Science Foundation prior to commencement (https://doi.org/10.17605/OSF.IO/BWJQ5). We performed a comprehensive search of Medline, EMBASE, and CINAHL in collaboration with a medical librarian, and we included any article published through July 31, 2021, using keywords relating to the TGD population, emergency medicine, and guidelines (Appendix B). A gray- literature search of Google Scholar and a focused search of relevant EM and TGD health societies were also completed for that timeline. We included articles if they represented a CPG, BPS, consensus document or other structured guidance for medical care for TGD populations of any age, in any practice setting, any large region, or nation, and if they were available in English.

Articles were excluded if they were narrative or systematic reviews, offered unstructured/non-medical guidance, if they were of local/municipal or single institution in scope, or if they were replaced by a more recent version of the guideline (Appendix B, Box C). Three reviewers independently screened title/abstracts and full text, and conflicts were resolved by group consensus. Included studies were reviewed by two independent reviewers in Covidence (covidence.org) and were analyzed for ED-relevant recommendations using a keyword search for “emergency.”

**Figure 1.**
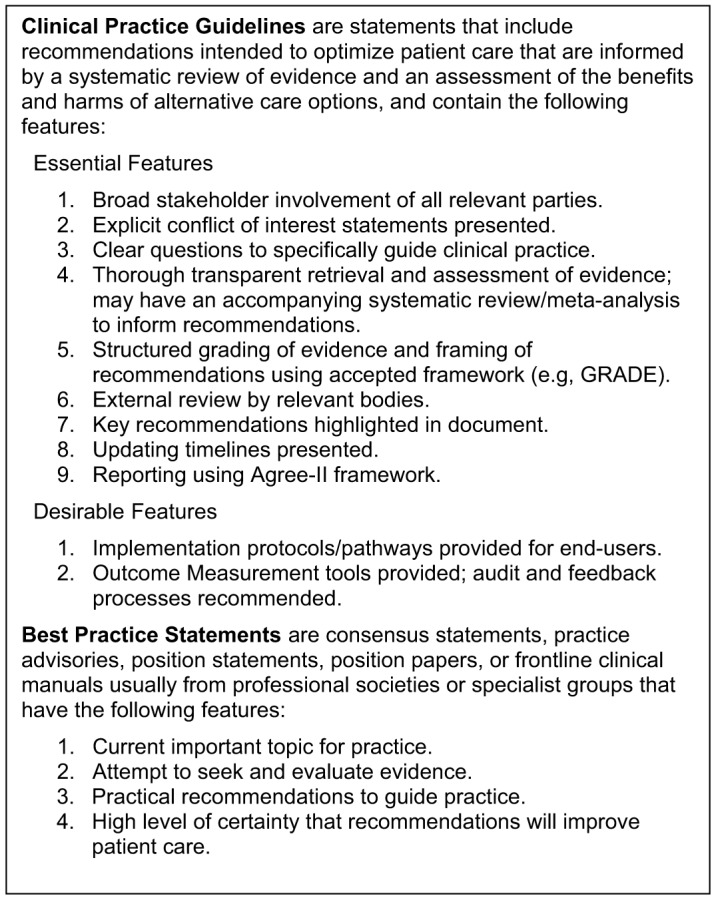
Key features of a clinical practice guideline or best practice statement.^14^ ^,^ ^15^ ^,^ ^19^ ^,^ ^21^

The individual recommendations relevant to the ED were coded as CPG or BPS using the criteria to be found in Figure 1 by country or region of origin. We defined “ED-relevance” as any recommendation pertaining to any process flow point during that ED visit: decision to come to ED; prehospital care; registration; triage; waiting room experience; rooming/initial nursing care; history and physical exam; investigations; diagnoses; treatment; disposition/discharge planning; and/or follow-up care. Three reviewers independently abstracted data with two reviewers per citation, and conflicts in coding were resolved by consensus. The data extraction template is available in Appendix B.

The methodological quality of included guidelines was evaluated using the AGREE-II instrument (four independent raters: AC, SKP, MK, SU), and individual EM-relevant recommendations with the AGREE-REX tool (three independent raters, AC, SKP, MK). Raters received training in instrument use via an online tutorial available through McMaster University, and from senior researchers on the project. We calculated rating tool scores using AGREE Trust calculator for AGREE-II (downloaded for free from the AGREE Trust website) and using Excel (Microsoft Corporation, Redmond, WA) for AGREE-REX using the calculations provided in the instrument manual. Using the interpretation suggestions in the original AGREE-II and AGREE-REX instruments^23^ ^,^ ^24^ a domain score <30% was considered low quality, a score of 30-70% was considered moderate quality, and over 70% was considered high quality. We assessed interrater reliability through use of the intraclass correlation coefficient (ICC) statistic using SPSS Statistics for Windows version 28.0 (IBM Corporation. Armonk, NY). An ICC score < 0.5 is considered poor, from 0.5– <0.75 moderate, from 0.75 to <.90 good, and >0.90 excellent.^25^

## RESULTS

**Figure 2.**
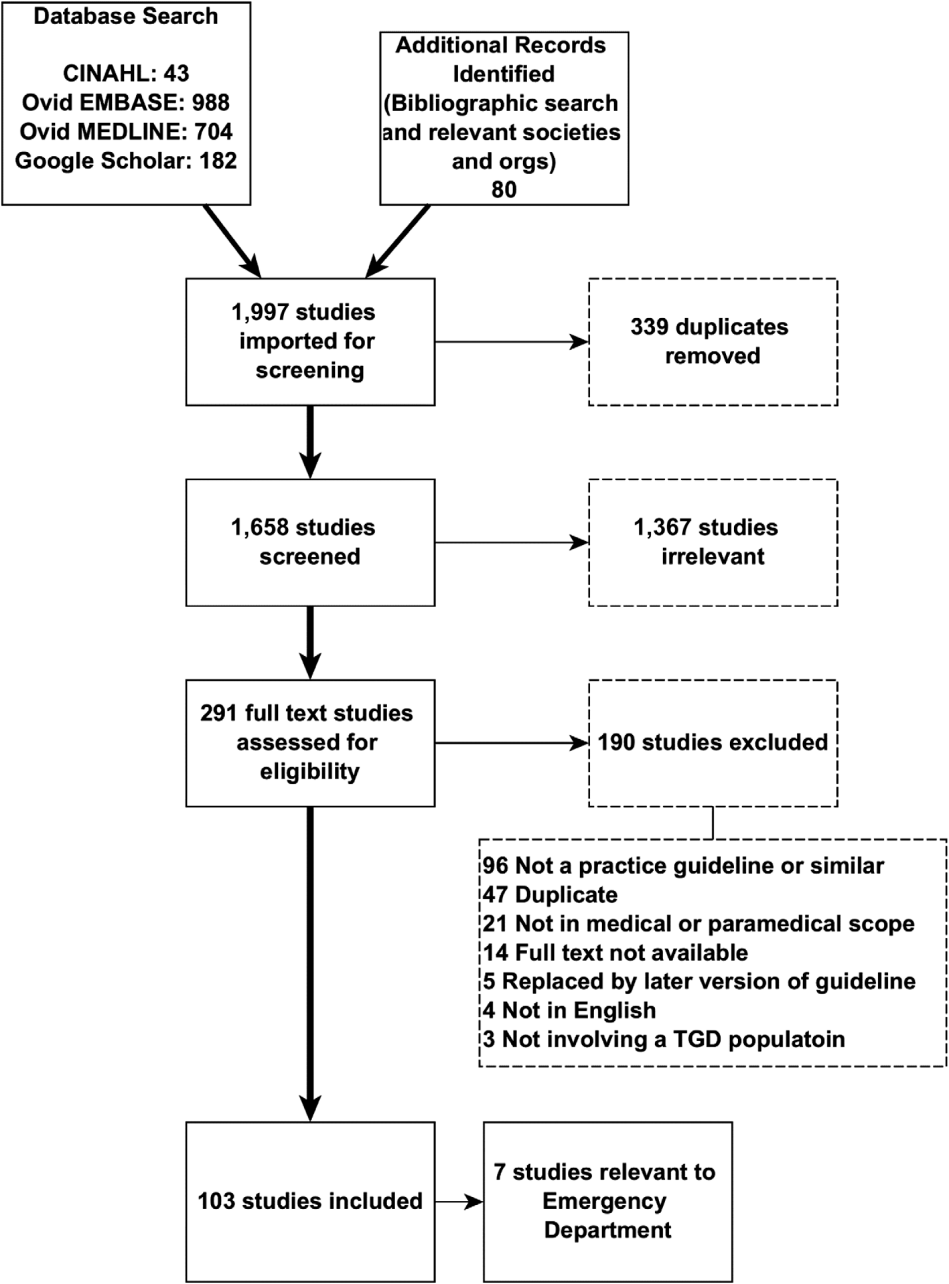
PRISMA diagram.

The literature search identified 1,997 articles, and 339 duplicates were removed. We screened titles and abstracts of 1,658 articles, with 1,367 not meeting inclusion criteria. Of the 291 articles undergoing full text review, 190 were excluded. Of the 103 remaining (Appendix A), seven articles were found to have 10 ED-relevant recommendations, and these were analysed using AGREE-II and AGREE-REX instruments. The literature search is summarized in the PRISMA flow diagram (Figure 2).

A summary of the appraised articles can be found in Table 1, Appendix C.^26^ ^–^ ^32^ Six of the articles met criteria as a CPG, and one as a BPS. Four of the articles were related to HIV care guidelines, one focused on comprehensive care of TGD populations, and two were focused on other minority populations, of which TGD people were a subset. The overall quality was judged by AGREE-II to be high in four of the articles.^26^ ^,^ ^29^ ^,^ ^30^ ^,^ ^32^

**Figure 3.**
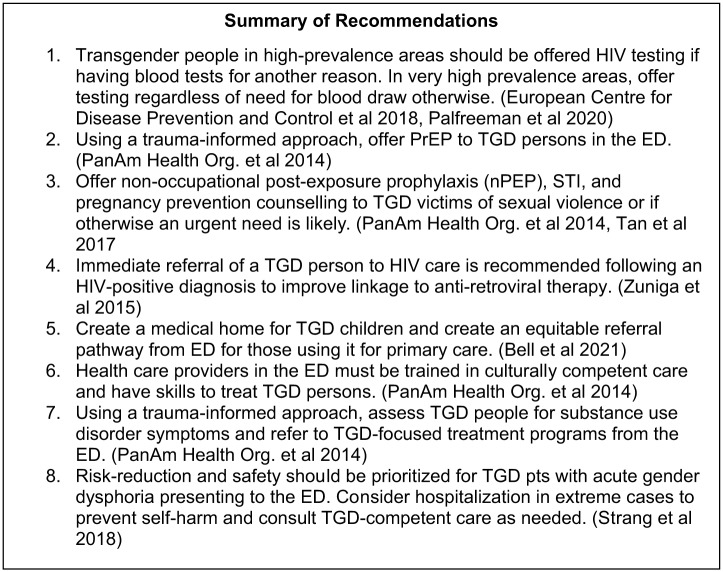
Summary of recommendations.^26^ ^–^ ^32^ *TGD*, transgender diverse; *ED*, emergency department; *PrEP*, pre-exposure prophylaxis; *TGD*, transgender diverse; *nPEP*, non-occupational post-exposure prophylaxis; *STI*, sexually transmitted infections; *ED*, emergency department.

The 10 individual recommendations relevant to ED care are summarized in Figure 3. A more detailed list with AGREE-REX evaluations can be found in Table 2, Appendix C. Overall, eight recommendations were considered high quality using AGREE-REX, with two having no consensus.

Interclass correlations for AGREE-II showed good correlation for scope and purpose, rigor of development, applicability, and editorial independence (Table 3, Appendix C). Stakeholder involvement showed moderate correlations, while clarity of presentation had poor ICC. The Agree-REX ICC was poor for values and preferences and ease of implementability but had good and moderate correlations for clinical applicability and total score overall, respectively (Table 4, Appendix C).

## DISCUSSION

This comprehensive systematic review of TGD patient care guidelines identified a small number of high-quality recommendations relevant to ED care. This represents the most comprehensive collection of guidance documents found to date, outpacing the previous guidelines (103 v 2–17).^16^ ^–^ ^18^ Seven were identified as either BPSs or CPGs with recommendations relevant to the ED. These guidelines were a mixture of general, multinational studies that provided higher level recommendations and improvements to care, along with country-specific studies that provided more targeted recommendations within the context of their healthcare structures. While the individual recommendations will not seem novel, this paper synthesizes the current collection of consensus documents for the care of TGD populations and sets the stage for development of future guidance products. There are currently actionable items for every ED to enhance the care of TGD people (summarized in Figure 3).

No recommendations pertaining to prehospital care, triage, waiting room, nursing, or follow-up care were identified. Key ED-relevant guidance focused on domains of ED attendance decisions, investigations, treatments, and disposition or discharge.

The general recommendations highlighted in this study focused on 1) HIV prevention, recommending that testing and referral services should be available and offered to TGD people; 2) cultural-competence training and trauma-informed approaches for TGD care provision, including adolescents in crisis; and 3) non-occupational post-exposure prophylaxis, recommending medications that should be readily available and included in situations of physical violence (see Appendix C Tables 1 and 2 for the specific guidelines and quality review). The more general guidelines focused on training and an equitable approach to care for emergency clinicians, but beyond training mandates they were not very specific in their implementation goals or skills requirements. We did not find any guidelines specifically oriented to the care of TGD people in the ED. From a quality standpoint, the evaluators scored most of the CPG/BPSs as high quality and the recommendations as applicable.

The strength of these recommendations is in their clarity regarding the testing and treatment of HIV for TGD populations in the ED and the need for comprehensive cultural-humility training and proper referral. They highlight the need for creating effective and equitable referral pathways for TGD patients of all ages presenting to the ED, and an opportunity to remove the barriers to care experienced by this population.^5^ ^,^ ^6^ ^,^ ^33^ The feasibility of HIV testing and referral from the ED is supported by recent systematic reviews, and universal implementation based on local prevalence is a reasonable goal.^34^ ^–^ ^36^ Therefore, implementation of these recommendations with meaningful community engagement is something EDs could achieve right away. It is also true that these recommendations can be applied universally to TGD and non-TGD people alike, and implementation of population-based screening should be very careful not to recapitulate stigmatizing TGD people as having higher inherent risk for HIV exposure.^35^ However, comprehensive clinical guidelines provide an opportunity to establish a standard of care in EDs and allow for TGD community stakeholder involvement to shape the urgent care this population needs.

The need for community engagement in primary research and knowledge translation, including guideline development, is critical for creating trustworthy and transparent guidance documents.^37^ In general, TGD populations and queer people have found the ED to be a de-valuing and discriminatory space, like much of medicine,^38^ and this has resulted in the disconnection between the needs of the community and the guidelines for care that have been largely created in a researcher/clinician-oriented manner.^21^ Purposeful community engagement models are needed to make any future guidelines relevant to the community and to remove barriers to ED care in all phases (decision to attend ED through discharge/follow-up). This comprehensive review identifies the current state of guidance literature for ED TGD care and highlights opportunities for improvement. For example, recommendations for equitable collection and use of gender identity information at triage,^39^ ^–^ ^41^ the safe use of names and pronouns,^4^ taking a sexual and gender history and organ inventory in TGD people,^42^ and an approach to surgical and medical complications for gender-affirming care^43^ are all ED-relevant questions that need to be integrated into good care for TGD populations.

To reinforce the need for community engagement, this review engaged members of the queer medical community in its production, and our group is developing one of the first diverse queer advisory panels to develop training systems for emergency clinicians. Our next step will be to broaden this into a national Delphi-type project to define the pathway for the next 10-year research program that will result in a comprehensive ED-focused guideline for all sexual and gender minorities, including TGD populations. This review, and ongoing similar reviews of sexual minorities and intersex populations, allows us to move onto community engagement so that we may draw patient-centered conclusions from these recommendations and produce more relevant community-focused recommendations in the form of a guideline.

## LIMITATIONS

Limitations in this study include inclusion of only English-language articles and a reliance on gray literature where guidelines are not published in standard databases. Thus, it is possible that we did not find relevant BPSs that may have augmented this review. At the time of the literature search, the World Professional Association of Transgender Health Standards of Care version 8 had not been released and so were not included. An informal review of this document found no ED-focused recommendations. As we were concerned with the application of the evidence to clinical care, we excluded systematic and narrative reviews from our analysis. It is possible that by excluding these two sources from our review of guidelines we are missing valuable information for emergency care; however, it becomes a challenge to integrate the very specific but sometimes inconclusive results from a systematic review or the very general conclusions from a narrative review, into discrete clinical practice without a consensus document to give them proper context. For this reason, we felt the risk of exclusion was not outweighed by the benefits of inclusion.

During rating of CPGs/BPSs, the poor ICC of evaluations of methodological domains was affected by the lack of readily available supplementary material that had more details about the methods of the guideline development, and if it was not included in the main paper it was judged as missing or not done. There was no ICC between assessments of values and preferences of stakeholders in the recommendations. This could be attributed to missing data in the main article, or due to differences in the understanding of the measure by the assessors. It may also be due to lack of overt statement of the values and preferences of the policy/decision makers and or guideline developers and the need to be inferred subjectively. As with the methods, the values and preferences statements were often published in supplemental material, leading to a more subjective assessment by reviewers.

Also, the absence of specific guidance for the ED is a strong limitation of this dataset and will require a more focused systematic review process to answer questions that arise out of the community consensus project mentioned above. The AGREE II process did include a risk of bias assessment (see section 9),^23^ but a more subtle form of research bias representing how guidelines are developed in general may not have been captured by this process. Some of the guidelines did include community engagement after the question generation and systematic review process but did not appear to involve community members in question prioritization. This suggests that all the included studies have a researcher-oriented bias that is not captured by the AGREE-II tool. Finally, the AGREE-REX tool suggests that five reviewers review each recommendation to increase reliability of the individual assessments; we had three independent reviewers, which may have decreased the reliability of our quality assessments.

## CONCLUSION

This is the most comprehensive review of clinical practice guidelines and best practice statements for ED care of transgender-diverse populations to date and reveals several important actionable recommendations for the care of TGD people in the emergency department. We identified opportunities for community-led development of a long-term research program and development of a comprehensive CPG for care of this population. Future endeavors should focus on creating ED-relevant guidance for culturally and medically competent care for TGD patients, with meaningful engagement of community members in all phases of developing guidance documents.

## Supplementary Information






