# Supplementary material for: Systematic Review, Quality Assessment, and Synthesis of Guidelines for Emergency Department Care of Transgender and Gender-diverse People: Recommendations for Immediate Action to Improve Care
Source: West J Emerg Med. 2023 Dec 20;25(1):94–100. doi: 10.5811/westjem.60632 (PMC10777181; doi:10.5811/westjem.60632)
Supplement: Supplementary file 1 [file wjem-25-94-s001.docx]

# Appendix A

## List of all included studies

1. Accelerating Change Transformation Team. *Feminizing Chest Surgery: Summary for Primary Care Providers |*. Alberta Medical Association; 2019. Accessed August 11, 2023. <https://actt.albertadoctors.org/media/domebfqf/feminizing-chest-surgery.pdf>

2. Accelerating Change Transformation Team. *Masculinizing Chest Surgery: Summary for Primary Care Providers*. Alberta Medical Association; 2019. Accessed August 11, 2023. <https://actt.albertadoctors.org/media/h41k5ev1/masculinizing-chest-surgery.pdf>

3. Accelerating Change Transformation Team. *Metoidioplasty: Summary for Primary Care Providers*. Alberta Medical Association; 2019. Accessed August 11, 2023. <https://actt.albertadoctors.org/media/uxklilte/metoidioplasty.pdf>

4. Accelerating Change Transformation Team. *Phalloplasty: Summary for Primary Care Providers*. Alberta Medical Association; 2019. Accessed August 11, 2023. <https://actt.albertadoctors.org/media/rjwbo1xo/phalloplasty.pdf>

5. Accelerating Change Transformation Team. *Transgender Health in Primary Care: Initial Assessment*. Alberta Medical Association; 2019. Accessed August 11, 2023. <https://actt.albertadoctors.org/media/or0fnwxz/initial-assessment-gender-dysphoria.pdf>

6. Accelerating Change Transformation Team. *Vaginoplasty: Summary for Primary Care Providers*. Alberta Medical Association; 2019. Accessed August 11, 2023. <https://actt.albertadoctors.org/media/rnthet0r/vaginoplasty.pdf>

7. Adelson SL. Practice Parameter on Gay, Lesbian, or Bisexual Sexual Orientation, Gender Nonconformity, and Gender Discordance in Children and Adolescents. *Journal of the American Academy of Child & Adolescent Psychiatry*. 2012;51(9):957-974. doi:[10.1016/j.jaac.2012.07.004](https://doi.org/10.1016/j.jaac.2012.07.004)

8. Álvarez LM. TGEU-Guidelines-to-Human-Rights-Based-Trans-specific-Healthcare-EN.pdf. Published online 2019. <https://tgeu.org/wp-content/uploads/2019/12/TGEU-Guidelines-to-Human-Rights-Based-Trans-specific-Healthcare-EN.pdf>

9. American College Health Association, Coalition of Allies for LGBT Health, Consortium of Higher Education LGBT Resource Professionals. ACHA Guidelines: Trans-inclusive college health programs. *Journal of American College Health*. 2016;64(2):162-164. doi:[10.1080/07448481.2016.1141017](https://doi.org/10.1080/07448481.2016.1141017)

10. American Geriatrics Society Ethics Committee. American Geriatrics Society care of lesbian, gay, bisexual, and transgender older adults position statement: American Geriatrics Society Ethics Committee. *Journal of the American Geriatrics Society*. 2015;63(3):423-426. doi:[10.1111/jgs.13297](https://doi.org/10.1111/jgs.13297)

11. Anderson RA, Amant F, Braat D, et al. ESHRE guideline: Female fertility preservation. *Human Reproduction Open*. 2021;2020(4). doi:[10.1093/hropen/hoaa052](https://doi.org/10.1093/hropen/hoaa052)

12. Anonymous. Standards of care: the hormonal and surgical sex reassignment of gender dysphoric persons. Harry Benjamin International Gender Dysphonia Association. *Archives of sexual behavior*. 1985;14(1):79-90.

13. Anonymous. Health risks and needs of lesbian, gay, bisexual, transgender, and questioning adolescents position statement. *Journal of pediatric health care*. 2011;25(6):A9-10.

14. Anonymous. ACOG Committee Opinion No. 749: Marriage and Family Building Equality for Lesbian, Gay, Bisexual, Transgender, Queer, Intersex, Asexual, and Gender Nonconforming Individuals. *Obstetrics and gynecology (New York 1953)*. 2018;132(2):e82-e86. doi:[10.1097/AOG.0000000000002765](https://doi.org/10.1097/AOG.0000000000002765)

15. Anonymous. ACOG Committee Opinion No. 758: Promoting Healthy Relationships in Adolescents. *Obstetrics and gynecology*. 2018;132(5):e213-e220. doi:[10.1097/AOG.0000000000002945](https://doi.org/10.1097/AOG.0000000000002945)

16. Anonymous. ACOG Committee Opinion No. 762: Prepregnancy Counseling. *Obstetrics and gynecology*. 2019;133(1):e78-e89. doi:[10.1097/AOG.0000000000003013](https://doi.org/10.1097/AOG.0000000000003013)

17. Anonymous. Preexposure prophylaxis for the prevention of HIV infection: Recommendation statement. *American Family Physician*. 2019;100(10):636F-636J.

18. Anonymous. Prepregnancy counseling: Committee Opinion No. 762. *Fertility and Sterility*. 2019;111(1):32-42. doi:[10.1016/j.fertnstert.2018.12.003](https://doi.org/10.1016/j.fertnstert.2018.12.003)

19. Becker S, Bosinski H, Wille R, et al. German standards for the treatment and diagnostic assessment of transsexuals. *International Journal of Transgenderism*. 1998;2(4). <http://www.symposion.com/ijt/ijtc0603.htm>

20. Bekker LG, Rebe K, Venter F, et al. Southern African guidelines on the safe use of pre-exposure prophylaxis in persons at risk of acquiring HIV-1 infection. *Southern African journal of HIV medicine*. 2016;17(1):455. doi:[10.4102/sajhivmed.v17i1.455](https://doi.org/10.4102/sajhivmed.v17i1.455)

21. Bell S, Deen JF, Fuentes M, Moore K, Committee on Native American Child Health. Caring for American Indian and Alaska Native Children and Adolescents. *Pediatrics*. 2021;147 done(4). doi:[10.1542/peds.2021-050498](https://doi.org/10.1542/peds.2021-050498)

22. Bhugra D, Gupta S, Schouler-Ocak M, et al. EPA Guidance mental health care of migrants. *European Psychiatry*. 2014;29(2):107-115. doi:[10.1016/j.eurpsy.2014.01.003](https://doi.org/10.1016/j.eurpsy.2014.01.003)

23. Bonnington A, Dianat S, Kerns J, et al. Society of Family Planning clinical recommendations: Contraceptive counseling for transgender and gender diverse people who were female sex assigned at birth. *Contraception*. 2020;102(2):70-82. doi:[10.1016/j.contraception.2020.04.001](https://doi.org/10.1016/j.contraception.2020.04.001)

24. Bourns A. *Guidelines for Gender-Affirming Primary Care with Trans and Non-Binary Patients, 4th Edition*. Rainbow Health Ontario; 2019. <https://www.rainbowhealthontario.ca/news-publications/rho-publications-resources/>

25. Bradley B, Kelts S, Robarge D, Davis C, Delger S, Compton L. NASN position statement: Sexual orientation and gender identity/expression (sexual minority students): school nurse practice. *NASN school nurse*. 2013;28(2):112-113.

26. Brady M, Rodger A, Asboe D, et al. BHIVA/BASHH guidelines on the use of HIV pre–exposure prophylaxis (PrEP) 2018. *HIV Medicine*. 2019;20(S2):s2-s80. doi:[10.1111/hiv.12718](https://doi.org/10.1111/hiv.12718)

27. Brook G, Church H, Evans C, et al. 2019 UK National Guideline for consultations requiring sexual history taking : Clinical Effectiveness Group British Association for Sexual Health and HIV. *International journal of STD & AIDS*. 2020;31(10):920-938. doi:[10.1177/0956462420941708](https://doi.org/10.1177/0956462420941708)

28. Buchting F, Margolies L, Bare M, et al. *LGBT Best and Promising Practices Throughout the Cancer Continuum*. LGBT HealthLink; 2015. <https://www.lgbthealthlink.org/Assets/U/Documents/Cancer-Best-Practices/cbpp-april2016.pdf>

29. Caceres CF, ed. *Guidelines: Prevention and Treatment of HIV and Other Sexually Transmitted Infections among Men Who Have Sex with Men and Transgender People: Recommendations for a Public Health Approach 2011. Geneva: WHO; 2011*. World Health Organization; 2011.

30. Cheung AS, Wynne K, Erasmus J, Murray S, Zajac JD. Position statement on the hormonal management of adult transgender and gender diverse individuals. *The Medical journal of Australia*. 2019;211(3):127-133. doi:[10.5694/mja2.50259](https://doi.org/10.5694/mja2.50259)

31. Chidiac C, Zakhem AE, Osman H, Yamout R. Recommendations to Improve Palliative Care Provision for Marginalized Populations: Summary of a Roundtable Discussion. *Journal of palliative medicine*. 2021;(9808462). doi:[10.1089/jpm.2020.0548](https://doi.org/10.1089/jpm.2020.0548)

32. Coleman E, Bockting W, Botzer M, et al. Standards of Care for the Health of Transsexual, Transgender, and Gender-Nonconforming People, Version 7. *International Journal of Transgenderism*. 2012;13(4):165-232. doi:[10.1080/15532739.2011.700873](https://doi.org/10.1080/15532739.2011.700873)

33. Committee On Adolescence. Office-based care for lesbian, gay, bisexual, transgender, and questioning youth. *Pediatrics*. 2013;132(1):198-203.

34. Committee on Gynecologic Practice and Committee on Health Care for Underserved Women. Health Care for Transgender and Gender Diverse Individuals: ACOG Committee Opinion, Number 823. *Obstetrics & Gynecology*. 2021;137(3):e75-e88. doi:[10.1097/AOG.0000000000004294](https://doi.org/10.1097/AOG.0000000000004294)

35. Cundill P. Hormone therapy for trans and gender diverse patients in the general practice setting. *Aust J Gen Pract*. 2020;49(7):385-390. doi:[10.31128/AJGP-01-20-5197](https://doi.org/10.31128/AJGP-01-20-5197)

36. Dahl M, Feldman JL, Goldberg JM, Jaberi A, Vancouver Coastal Health. Endocrine Therapy for Transgender Adults in British Columbia: Suggested Guidelines: Physical Aspects of Transgender Endocrine Therapy. Published online 2015. <http://www.phsa.ca/transcarebc/Documents/HealthProf/BC-Trans-Adult-Endocrine-Guidelines-2015.pdf>

37. Daniel H, Butkus R, Health and Public Policy Committee of American College of Physicians. Lesbian, Gay, Bisexual, and Transgender Health Disparities: Executive Summary of a Policy Position Paper From the American College of Physicians. *Annals of internal medicine*. 2015;163(2):135-137. doi:[10.7326/M14-2482](https://doi.org/10.7326/M14-2482)

38. Davidson A, Franicevich J, Freeman M, et al. *Tom Waddell Health Center*.; 2013. <http://www.dph.sf.ca.us/chn/HlthCtrs/transgender.htm>

39. de Vries ALC, Klink D, Cohen-Kettenis PT. What the Primary Care Pediatrician Needs to Know About Gender Incongruence and Gender Dysphoria in Children and Adolescents. *Pediatric clinics of North America*. 2016;63(6):1121-1135. doi:[10.1016/j.pcl.2016.07.011](https://doi.org/10.1016/j.pcl.2016.07.011)

40. Deutsch MB. *Terminology and Definitions, Guidelines for the Primary and Gender-Affirming Care of Transgender and Gender Nonbinary People*. USCF Center of Excellence for Transgender Health; 2016.

41. Deutsch MB. *Guidelines for the Primary and Gender-Affirming Care of Transgender and Gender Nonbinary People*. UCSF Center of Excellence for Transgender Health; 2016.

42. Deutsch MB, Green J, Keatley J, et al. Electronic medical records and the transgender patient: recommendations from the World Professional Association for Transgender Health EMR Working Group. *Journal of the American Medical Informatics Association : JAMIA*. 2013;20(4):700-703. doi:[10.1136/amiajnl-2012-001472](https://doi.org/10.1136/amiajnl-2012-001472)

43. Dunn P, Scout, Taylor JS. *Guidelines for Care of Lesbian, Gay, Bisexual, and Transgender Patients. 2006*. Gay and Lesbian Medical Association (GLMA); 2019.

44. Dwyer AA, Greenspan DL. Endocrine Nurses Society Position Statement on Transgender and Gender Diverse Care. *Journal of the Endocrine Society*. 2021;5(8). doi:[10.1210/jendso/bvab105](https://doi.org/10.1210/jendso/bvab105)

45. ESHRE Guideline Group on Female Fertility Preservation, Anderson RA, Amant F, et al. ESHRE guideline: female fertility preservation. *Human reproduction open*. 2020;2020(4). doi:[10.1093/hropen/hoaa052](https://doi.org/10.1093/hropen/hoaa052)

46. Esteva de Antonio I, Asenjo Araque N, Hurtado Murillo F, et al. Position statement: Gender dysphoria in childhood and adolescence. Working Group on Gender Identity and Sexual Development of the Spanish Society of Endocrinology and Nutrition (GIDSEEN). *Endocrinologia y nutricion : organo de la Sociedad Espanola de Endocrinologia y Nutricion*. 2015;62(8):380-383. doi:[10.1016/j.endonu.2015.03.004](https://doi.org/10.1016/j.endonu.2015.03.004)

47. European Centre for Disease Prevention and Control, Desai S, Hoekstra M, et al. *Public Health Guidance on HIV, Hepatitis B and C Testing in the EU/EEA : An Integrated Approach*. European Centre for Disease Prevention and Control; 2019. doi:[10.2900/79127](https://doi.org/10.2900/79127)

48. Ezra J. *Taking Charge: A Handbook for Health Care and Social Service Providers Working with Trans People*. (Fugler O, ed.). Trans Health Network of Quebec; 2011. Accessed August 11, 2023. <https://coco-net.org/wp-content/uploads/2013/03/TakingChargeHandbookupdated.pdf>

49. Fadus M, Hung K, Casoy F. Care Considerations for LGBTQ Patients in Acute Psychiatric Settings. *Focus*. 2020;18(3):285-288. doi:[10.1176/appi.focus.20200002](https://doi.org/10.1176/appi.focus.20200002)

50. Feldman J, Deutsch MB. Primary care of transgender individuals - UpToDate. Up To Date. Published 2023. Accessed August 11, 2023. <https://www.uptodate.com/contents/primary-care-of-transgender-individuals>

51. Feldman J, Goldberg J. *Transgender Primary Medical Care: Suggested Guidelines for Clinicians in British Columbia. Vancouver*. Vancouver Coastal Health, Transcend Transgender Support & Education Society, and Canadian Rainbow Health Coalition; 2006.

52. Ferri RL, Rosen-Carole CB, Jackson J, Carreno-Rijo E, Greenberg KB, Academy of Breastfeeding Medicine. ABM Clinical Protocol #33: Lactation Care for Lesbian, Gay, Bisexual, Transgender, Queer, Questioning, Plus Patients. *Breastfeeding medicine : the official journal of the Academy of Breastfeeding Medicine*. 2020;15(5):284-293. doi:[10.1089/bfm.2020.29152.rlf](https://doi.org/10.1089/bfm.2020.29152.rlf)

53. Fisher AD, Ristori J, Bandini E, et al. Medical treatment in gender dysphoric adolescents endorsed by SIAMS-SIE-SIEDP-ONIG. *Journal of endocrinological investigation*. 2014;37(7):675-687. doi:[10.1007/s40618-014-0077-6](https://doi.org/10.1007/s40618-014-0077-6)

54. Francis C, Grober E, Krakowsky Y, Potter E, Blodgett N. A Simple Guide for Simple Orchiectomy in Transition-Related Surgeries. *Sexual Medicine Reviews*. 2020;8(3):492-496. doi:[10.1016/j.sxmr.2019.11.004](https://doi.org/10.1016/j.sxmr.2019.11.004)

55. Gamble RM, Taylor SS, Huggins AD, Ehrenfeld JM. Trans-specific Geriatric Health Assessment (TGHA): An inclusive clinical guideline for the geriatric transgender patient in a primary care setting. *Maturitas*. 2020;132(7807333):70-75. doi:[10.1016/j.maturitas.2019.12.005](https://doi.org/10.1016/j.maturitas.2019.12.005)

56. Godano A, Maggi M, Jannini E, et al. SIAMS-ONIG Consensus on hormonal treatment in gender identity disorders. *Journal of endocrinological investigation*. 2009;32(10):857-864.

57. Graves L, Carson G, Poole N, et al. Guideline No. 405: Screening and Counselling for Alcohol Consumption During Pregnancy. *Journal of Obstetrics and Gynaecology Canada*. 2020;42(9):1158. doi:[10.1016/j.jogc.2020.03.002](https://doi.org/10.1016/j.jogc.2020.03.002)

58. Griggs J, Maingi S, Blinder V, et al. American Society of Clinical Oncology Position Statement: Strategies for Reducing Cancer Health Disparities Among Sexual and Gender Minority Populations. *Journal of clinical oncology : official journal of the American Society of Clinical Oncology*. 2017;35(19):2203-2208. doi:[10.1200/JCO.2016.72.0441](https://doi.org/10.1200/JCO.2016.72.0441)

59. Hadland SE, Yehia BR, Makadon HJ. Caring for Lesbian, Gay, Bisexual, Transgender, and Questioning Youth in Inclusive and Affirmative Environments. *Pediatric Clinics of North America*. 2016;63(6):955-969. doi:[10.1016/j.pcl.2016.07.001](https://doi.org/10.1016/j.pcl.2016.07.001)

60. Health Policy Project, Asia Pacific Transgender Networ, United Nations Development Programme. *Blueprint for the Provision of Comprehensive Care for Trans People and Trans Communities in Asia and the Pacific.* Futures Group, Health Policy Project; 2015.

61. Hembree WC, Cohen-Kettenis PT, Gooren L, et al. Endocrine Treatment of Gender-Dysphoric/Gender-Incongruent Persons: An Endocrine Society Clinical Practice Guideline. *The Journal of clinical endocrinology and metabolism*. 2017;102(11):3869-3903. doi:[10.1210/jc.2017-01658](https://doi.org/10.1210/jc.2017-01658)

62. Hsu KK, Marcell AV. CDC STD TREATMENT GUIDELINES... [including commentary by Elizabeth Miller]. *Contemporary Pediatrics*. 2015;32(11):28-28.

63. Janeway H, Coli CJ. Emergency care for transgender and gender-diverse children and adolescents. *Pediatric emergency medicine practice*. 2020;17(9):1-20.

64. Kendig NE, Cubitt A, Moss A, Sevelius J. Developing Correctional Policy, Practice, and Clinical Care Considerations for Incarcerated Transgender Patients Through Collaborative Stakeholder Engagement. *Journal of correctional health care : the official journal of the National Commission on Correctional Health Care*. 2019;25(3):277-286. doi:[10.1177/1078345819857113](https://doi.org/10.1177/1078345819857113)

65. Keuroghlian AS, Shtasel D, Bassuk EL. Out on the street: a public health and policy agenda for lesbian, gay, bisexual, and transgender youth who are homeless. *The American journal of orthopsychiatry*. 2014;84(1):66-72. doi:[10.1037/h0098852](https://doi.org/10.1037/h0098852)

66. Klein DA, Paradise SL, Goodwin ET. Caring for Transgender and Gender-Diverse Persons: What Clinicians Should Know. *AFP*. 2018;98(11):645-653.

67. Lamont J, Bajzak K, Bouchard C, et al. No. 279-Female Sexual Health Consensus Clinical Guidelines. *Journal of Obstetrics and Gynaecology Canada*. 2018;40(6):e451-e503. doi:[10.1016/j.jogc.2018.04.009](https://doi.org/10.1016/j.jogc.2018.04.009)

68. Lapinski J, Covas T, Perkins JM, et al. Best Practices in Transgender Health: A Clinician’s Guide. *Primary care*. 2018;45(4):687-703. doi:[10.1016/j.pop.2018.07.007](https://doi.org/10.1016/j.pop.2018.07.007)

69. Lavin A, LaMonte Askew G, Baum R, et al. Runaway youth: Caring for the nation’s largest segment of missing children. *Pediatrics*. 2020;145(2). doi:[10.1542/peds.2019-3752](https://doi.org/10.1542/peds.2019-3752)

70. Lopez X, Marinkovic M, Eimicke T, Rosenthal SM, Olshan JS, Pediatric Endocrine Society Transgender Health Special Interest Group. Statement on gender-affirmative approach to care from the pediatric endocrine society special interest group on transgender health. *Current opinion in pediatrics*. 2017;29(4):475-480. doi:[10.1097/MOP.0000000000000516](https://doi.org/10.1097/MOP.0000000000000516)

71. Maas AHEM, Rosano G, Cifkova R, et al. Cardiovascular health after menopause transition, pregnancy disorders, and other gynaecologic conditions: a consensus document from European cardiologists, gynaecologists, and endocrinologists. *European heart journal*. 2021;42(10):967-984. doi:[10.1093/eurheartj/ehaa1044](https://doi.org/10.1093/eurheartj/ehaa1044)

72. Maingi S, Bagabag AE, O’Mahony S. Current Best Practices for Sexual and Gender Minorities in Hospice and Palliative Care Settings. *Journal of pain and symptom management*. 2018;55(5):1420-1427. doi:[10.1016/j.jpainsymman.2017.12.479](https://doi.org/10.1016/j.jpainsymman.2017.12.479)

73. Majumder A, Chatterjee S, Maji D, et al. IDEA Group Consensus Statement on Medical Management of Adult Gender Incongruent Individuals Seeking Gender Reaffirmation as Female. *Indian journal of endocrinology and metabolism*. 2020;24(2):128-135. doi:[10.4103/ijem.IJEM_593_19](https://doi.org/10.4103/ijem.IJEM_593_19)

74. Martinez F. Update on fertility preservation from the Barcelona International Society for Fertility Preservation-ESHRE-ASRM 2015 expert meeting: indications, results and future perspectives. *Human reproduction (Oxford, England)*. 2017;32(9):1802-1811. doi:[10.1093/humrep/dex218](https://doi.org/10.1093/humrep/dex218)

75. McNamara MC, Ng H. Best practices in LGBT care: A guide for primary care physicians. *Cleveland Clinic journal of medicine*. 2016;83(7):531-541. doi:[10.3949/ccjm.83a.15148](https://doi.org/10.3949/ccjm.83a.15148)

76. Moreno-Perez O, Esteva De Antonio I. Clinical Practice Guidelines for Assessment and Treatment of Transsexualism. SEEN Identity and Sexual Differentiation Group (GIDSEEN). *Endocrinologia y Nutricion*. 2012;59(6):367-382. doi:[10.1016/j.endonu.2012.02.001](https://doi.org/10.1016/j.endonu.2012.02.001)

77. Muller A. *Sexual and Reproductive Health for Transgender and Gender Non-Conforming People: Guidelines for Healthcare Workers in Primary Care*. Cape Town: Gender Dynamix; 2013.

78. Oliphant J, Veale J, Macdonald J, et al. Guidelines for Gender Affirming Healthcare for Gender Diverse and Transgender Children, Young People and Adults in Aotearoa, New Zealand. *The New Zealand medical journal*. 2018;131(1487):86-96.

79. Palfreeman A, Sullivan A, Rayment M, et al. British HIV Association/British Association for Sexual Health and HIV/British Infection Association adult HIV testing guidelines 2020. *HIV Medicine*. 2020;21(S6):1-26. doi:[10.1111/hiv.13015](https://doi.org/10.1111/hiv.13015)

80. Pan American Health Organization, Bockting W, Keatley J, World Professional Association for Transgender Health. Blueprint for the Provision of Comprehensive Care for Trans Persons and Their Communities in the Caribbean and Other Anglophone Countries. Published online 2014. <https://www.paho.org/hq/dmdocuments/2014/2014-cha-blueprint-comprehensive-anglo-countries.pdf>

81. Primary Care Working Group. Gender-affirming Care for Trans, Two-Spirit, and Gender Diverse Patients in BC: A Primary Care Toolkit. Published online 2021.

82. Radix A, Meacher P, Anthony V, et al. *Protocols for the Provision of Hormone Therapy*. Callen-Lorde Community Health Centre; 2020. <https://callen-lorde.org/transhealth/callen-lorde-tgnc-hormone-therapy-protocols/>

83. Ralph D, Gonzalez-Cadavid N, Mirone V, et al. Trauma, gender reassignment, and penile augmentation. *The journal of sexual medicine*. 2010;7(4 Pt 2):1657-1667. doi:[10.1111/j.1743-6109.2010.01781.x](https://doi.org/10.1111/j.1743-6109.2010.01781.x)

84. Riechardt S, Waterloos M, Lumen N, et al. European Association of Urology Guidelines on Urethral Stricture Disease Part 3: Management of Strictures in Females and Transgender Patients. *European Urology Focus*. Published online 2021. doi:[10.1016/j.euf.2021.07.013](https://doi.org/10.1016/j.euf.2021.07.013)

85. Rosen HN, Hamnvik OPR, Jaisamrarn U, et al. Bone Densitometry in Transgender and Gender Non-Conforming (TGNC) Individuals: 2019 ISCD Official Position. *Journal of clinical densitometry : the official journal of the International Society for Clinical Densitometry*. 2019;22(4):544-553. doi:[10.1016/j.jocd.2019.07.004](https://doi.org/10.1016/j.jocd.2019.07.004)

86. Royal College of General Practitioners. *Guidelines for the Care of Trans Patients in Primary Care*. Royal College of General Practitioners; 2017. Accessed August 11, 2023. <https://www.rcgp.org.uk/getmedia/18e6238d-6fff-43c7-b027-e3fb2d718fff/RCGPNI-Trans-Patient-Guidelines-for-GPs-2017.pdf>

87. Shuhart CR, Yeap SS, Anderson PA, et al. Executive Summary of the 2019 ISCD Position Development Conference on Monitoring Treatment, DXA Cross-calibration and Least Significant Change, Spinal Cord Injury, Peri-prosthetic and Orthopedic Bone Health, Transgender Medicine, and Pediatrics. *Journal of clinical densitometry : the official journal of the International Society for Clinical Densitometry*. 2019;22(4):453-471. doi:[10.1016/j.jocd.2019.07.001](https://doi.org/10.1016/j.jocd.2019.07.001)

88. Society for Adolescent Health and Medicine. Recommendations for promoting the health and well-being of lesbian, gay, bisexual, and transgender adolescents: a position paper of the Society for Adolescent Health and Medicine. *The Journal of adolescent health : official publication of the Society for Adolescent Medicine*. 2013;52(4):506-510. doi:[10.1016/j.jadohealth.2013.01.015](https://doi.org/10.1016/j.jadohealth.2013.01.015)

89. Society for Adolescent Health and Medicine. Promoting Health Equality and Nondiscrimination for Transgender and Gender-Diverse Youth. *The Journal of adolescent health : official publication of the Society for Adolescent Medicine*. 2020;66(6):761-765. doi:[10.1016/j.jadohealth.2020.03.016](https://doi.org/10.1016/j.jadohealth.2020.03.016)

90. Strang JF, Meagher H, Kenworthy L, et al. Initial Clinical Guidelines for Co-Occurring Autism Spectrum Disorder and Gender Dysphoria or Incongruence in Adolescents. *Journal of Clinical Child & Adolescent Psychology*. 2018;47(1):105-115. doi:[10.1080/15374416.2016.1228462](https://doi.org/10.1080/15374416.2016.1228462)

91. T’Sjoen G, Arcelus J, De Vries ALC, et al. European Society for Sexual Medicine Position Statement “Assessment and Hormonal Management in Adolescent and Adult Trans People, With Attention for Sexual Function and Satisfaction”. *The journal of sexual medicine*. 2020;17(4):570-584. doi:[10.1016/j.jsxm.2020.01.012](https://doi.org/10.1016/j.jsxm.2020.01.012)

92. Tan DHS, Hull MW, Yoong D, et al. Canadian guideline on HIV pre-exposure prophylaxis and nonoccupational postexposure prophylaxis. *CMAJ*. 2017;189 (DONE)(47):E1448-E1458. doi:[10.1503/cmaj.170494](https://doi.org/10.1503/cmaj.170494)

93. Telfer MM, Tollit MA, Pace CC, Pang KC. Australian standards of care and treatment guidelines for transgender and gender diverse children and adolescents. *The Medical journal of Australia*. 2018;209(3):132-136.

94. Thompson J, Hopwood R, deNormand S, Cavanaugh T. *Medical Care of Trans and Gender Diverse Adults*. Fenway Health; 2021. <https://www.lgbtqiahealtheducation.org/wp-content/uploads/2021/07/Medical-Care-of-Trans-and-Gender-Diverse-Adults-Spring-2021.pdf>

95.Thompson MA, Horberg MA, Agwu AL, et al. Primary Care Guidance for Persons With Human Immunodeficiency Virus: 2020 Update by the HIV Medicine Association of the Infectious Diseases Society of America. *Clinical infectious diseases*. Published online 2020. doi:[10.1093/cid/ciaa1391](https://doi.org/10.1093/cid/ciaa1391)

96. Unknown. *Guidelines: Creating a Safe Clinical Environment for Lesbian, Gay, Bisexual, Transgender, and Intersex (LGBTI) Patients*. Gay and Lesbian Medical Association; 2005.

97. Workowski KA, Bolan GA, Centers for Disease Control and Prevention. Sexually transmitted diseases treatment guidelines, 2015. *MMWR Recommendations and reports*. 2015;64(RR-03):1-137.

98. World Health Organization. *Guidance on Pre-Exposure Oral Prophylaxis (PrEP) for Serodiscordant Couples, Men and Transgender Women Who Have Sex with Men at High Risk of HIV: Recommendations for Use in the Context of Demonstration Projects*. World Health Organization; 2012. <http://www.ncbi.nlm.nih.gov/books/NBK132003/>

99. World Health Organization. *Consolidated Guidelines on HIV Prevention, Testing, Treatment, Service Delivery and Monitoring: Recommendations for a Public Health Approach*. World Health Organization; 2021. <https://apps.who.int/iris/handle/10665/342899>

100. Wright E, Grulich A, Roy K, et al. Australasian Society for HIV, Viral Hepatitis and Sexual Health Medicine HIV pre-exposure prophylaxis: clinical guidelines. Update April 2018. *Journal of virus eradication*. 2018;4(3):143-159.

101. Wylie K, Barrett J, Besser M, et al. Good Practice Guidelines for the Assessment and Treatment of Adults with Gender Dysphoria. *Sexual & Relationship Therapy*. 2014;29(2):154-214. doi:[10.1080/14681994.2014.883353](https://doi.org/10.1080/14681994.2014.883353)

102. Yen-Hao Chu I, Wen-Wei Ku S, Li CW, et al. Taiwan guideline on oral pre-exposure prophylaxis for HIV prevention - 2018 update. *Journal of microbiology, immunology, and infection = Wei mian yu gan ran za zhi*. 2020;53(1):1-10. doi:[10.1016/j.jmii.2019.09.003](https://doi.org/10.1016/j.jmii.2019.09.003)

103. Zuniga JM, Bekker LG, Montaner J, et al. IAPAC guidelines for optimizing the HIV care continuum for adults and adolescents. *Journal of the International Association of Providers of AIDS Care*. 2015;14((Zuniga) International Association of Providers of AIDS Care, 1990 M Street NW, Washington, DC 20036, United States):S3-S34. doi:[10.1177/2325957415613442](https://doi.org/10.1177/2325957415613442)
