# Supplementary material for: Systematic Review, Quality Assessment, and Synthesis of Guidelines for Emergency Department Care of Transgender and Gender-diverse People: Recommendations for Immediate Action to Improve Care
Source: West J Emerg Med. 2023 Dec 20;25(1):94–100. doi: 10.5811/westjem.60632 (PMC10777181; doi:10.5811/westjem.60632)
Supplement: Supplementary file 2 [file wjem-25-94-s002.docx]

# Appendix B

## Example of Search Strategy

Ovid MEDLINE(R) ALL <1946 to July 08, 2022>

1 exp clinical pathway/

2 exp clinical protocol/

3 exp consensus/

4 exp consensus development conference/

5 exp consensus development conferences as topic/

6 critical pathways/

7 exp guideline/

8 guidelines as topic/

9 exp practice guideline/

10 practice guidelines as topic/

11 health planning guidelines/

12 (guideline or practice guideline or consensus development conference or consensus development conference, NIH).pt.

13 (position statement* or policy statement* or practice parameter* or best practice*).ti,ab,kf,kw.

14 (standards or guideline or guidelines).ti,kf,kw.

15 ((practice or treatment* or clinical) adj guideline*).ab.

16 (CPG or CPGs).ti.

17 consensus*.ti,kf,kw.

18 consensus*.ab. /freq=2

19 ((critical or clinical or practice) adj2 (path or paths or pathway or pathways or protocol*)).ti,ab,kf,kw.

20 recommendat*.ti,kf,kw.

21 (care adj2 (standard or path or paths or pathway or pathways or map or maps or plan or plans)).ti,ab,kf,kw.

22 (algorithm* adj2 (screening or examination or test or tested or testing or assessment* or diagnosis or diagnoses or diagnosed or diagnosing)).ti,ab,kf,kw.

23 (algorithm* adj2 (pharmacotherap* or chemotherap* or chemotreatment* or therap* or treatment* or intervention*)).ti,ab,kf,kw.

24 1 or 2 or 3 or 4 or 5 or 6 or 7 or 8 or 9 or 10 or 11 or 12 or 13 or 14 or 15 or 16 or 17 or 18 or 19 or 20 or 21 or 22 or 23

25 "gender minorities"/

26 gender minor*.mp.

27 transgender*.mp.

28 trans gender*.mp.

29 transsexual*.mp.

30 trans sexual*.mp.

31 transfeminine*.mp.

32 trans feminine*.mp.

33 transmasculin*.mp.

34 trans masculin*.mp.

35 transvestite*.mp.

36 gender non conforming.mp.

37 gender noncomforming.mp.

38 gender non binary.mp.

39 gender nonbinary.mp.

40 25 or 26 or 27 or 28 or 29 or 30 or 31 or 32 or 33 or 34 or 35 or 36 or 37 or 38 or 39

44 24 and 40 1

45 limit 44 to english language

46 limit 44 to animals

47 45 not 46

## Inclusion/Exclusion Criteria

Box C: Inclusion Exclusion Criteria

**Inclusion Criteria**

1. Any clinical practice guidelines, best practice statement, consensus document, critical pathway, or other systematic documents outlining holistic standard of care recommendations for transgender or gender non-binary/nonconforming persons.

2. Medical or para-medical in scope.

3. In any setting, community or hospital.

4. In English.

5. Any region or nation.

6. Of national, or international in scope (see note in exclusion criteria).

**Exclusion Criteria**

1. Review articles, including narrative reviews or general overviews of trans care that do not make systematic clinical recommendations or are focused on one clinical question.

2. Experimental or observational studies, editorials, or letters to the editor.

3. Of regional or single hospital system or community in scope (a provincial or state guideline that is applicable to those outside the state or nationally is acceptable, a local policy or guidance document meant for a local audience only is not).

4. Non-medical guidelines.

5. If a more recent version of the guidelines exists, then the earlier version will be excluded.

## Data Extraction Template

Study ID:

Title:

Author and Year Published:

Country/Region in which document is relevant:

- United States
- United Kingdom
- Canada
- Australia
- North America
- Caribbean Islands
- South America
- Europe
- Africa
- Asia
- International
- Other: ____________

Source

- Peer-reviewed article
- Non-peer reviewed article

Does the document include recommendations for care in the Emergency Department?

- Yes
- No

If Yes to above, what stage of care does it concern, and what is/are the recommendation(s)?

- Decision to Come to ED: ____
- Prehospital Care: ____
- Registration: ____
- Triage: ____
- Waiting Room: ____
- Rooming/Initial Nursing Care: ____
- History and Physical exam: ____
- Investigations: ____
- Diagnoses : ____
- Treatment : ____
- Disposition/Discharge Planning: ____
- Follow-up care: ____

Does this meet the definition of a clinical practice guideline or one of a best practice statement?

- Clinical Practice Guideline
- Best Practice Statement
