# Supplementary material for: Systematic Review, Quality Assessment, and Synthesis of Guidelines for Emergency Department Care of Transgender and Gender-diverse People: Recommendations for Immediate Action to Improve Care
Source: West J Emerg Med. 2023 Dec 20;25(1):94–100. doi: 10.5811/westjem.60632 (PMC10777181; doi:10.5811/westjem.60632)
Supplement: Supplementary file 3 [file wjem-25-94-s003.docx]

# Systematic Review, Quality Assessment, and Synthesis of Guidelines for Emergency Department Care of Transgender and Gender Diverse People: recommendations for immediate action to improve care

# **Appendix C**

Table 1 Included Studies with AGREE II Scores

| Author and Year | **Title** | **Country or Region** | **CPG or BPS** | **Dom. 1 (%) Scope** | **Dom. 2 (%) Stake.** | **Dom. 3 (%) Rigor** | **Dom. 4 (%) Clarity** | **Dom. 5 (%) Applic.** | **Dom. 6 (%) Editor.** | **Overall Score** | **Overall Quality** |
| --- | --- | --- | --- | --- | --- | --- | --- | --- | --- | --- | --- |
|  |  |  |  |  |  |  |  |  |  |  |  |
| Bell et al 2021 | Caring for American Indian and Alaska Native Children and Adolescents. | USA | BPS | 65 | 44 | 9 | 65 | 18 | 90 | 21 | Low |
| European Centre for Disease Prevention and Control et al 2018 | Public health guidance on HIV, hepatitis B and C testing in the EU/EEA: an integrated approach. | Europe | CPG | 89 | 65 | 58 | 82 | 68 | 42 | 75 | High |
| Palfreeman et al 2020 | British HIV Assoc./British Assoc. for Sexual Health and HIV/British Infection Assoc. Adult HIV testing guidelines 2020 | United Kingdom | CPG | 88 | 76 | 77 | 94 | 63 | 73 | 83 | High |
| Pan American Health Org. et al 2014 | Blueprint for the Provision of Comprehensive Care for Trans Persons and Their Communities in the Caribbean and Other Anglophone Countries | Caribbean | CPG | 88 | 79 | 16 | 79 | 46 | 38 | 38 | Moderate |
| Strang et al 2018 | Initial Clinical Guidelines for Co-Occurring Autism Spectrum Disorder and Gender Dysphoria or Incongruence in Adolescents | USA | CPG | 83 | 49 | 38 | 67 | 18 | 21 | 42 | Moderate |
| Tan et al 2017 | Canadian guideline on HIV pre-exposure prophylaxis and nonoccupational postexposure prophylaxis | Canada | CPG | 99 | 69 | 76 | 96 | 57 | 85 | 83 | High |
| Zuniga et al 2015 | IAPAC guidelines for optimizing the HIV care continuum for adults and adolescents | Internat. | CPG | 79 | 51 | 51 | 89 | 58 | 92 | 71 | High |

CPG= clinical practice guideline, BPS = best practice statement.

Table 2: Individual Recommendations and AGREE-REX Ratings

| Author Year | Recommendation | ED Visit Event | Domain 1 (%)  Clinical Applicability | Domain 2 (%)  Values and  Preferences | Domain 3 (%)  Implementability | Total (%) | Recommend Use?  (Yes/Yes with reservations/No) |
| --- | --- | --- | --- | --- | --- | --- | --- |
| European Centre for Disease Prevention and Control et al 2018 | Transgender people in high-prevalence areas should be offered HIV testing if having blood tests for another reason. | Investigations | 35 | 15 | 39 | 27 | No Consensus |
| Bell et al 2021 | Create a medical home sensitive to discrimination against TGD people. Work with ED’s to create referral pathway from ED for those who use ED for primary care. | Decision to come to ED | 76 | 29 | 56 | 51 | Yes with reservations |
| Palfreeman et al 2020 | For TGD people in high and very high prevalence areas offer HIV testing when taking blood for other reasons. In extremely high prevalence areas offer testing regardless of routine venipuncture. | Investigations | 74 | 44 | 81 | 62 | Yes with reservations |
| Pan American Health Org. et al 2014 | Health care providers in the ED must be trained in culturally competent care and have skills to treat TGD persons. | Decision to come to ED | 54 | 31 | 67 | 46 | No consensus |
|  | Assess TGD people for substance use disorder Sx and refer to TGD-focused treatment programs from the ED. Providers need to be respectful and aware of a potential traumatic history. | History and Physical | 31 | 21 | 58 | 33 | Yes |
|  | If appropriate, offer pre-exposure prophylaxis for HIV. Health care providers in ED’s must have skills to deal with TGD persons. Use standardized approach to aggressive pts with trauma informed approach. | Investigations | 54 | 42 | 64 | 51 | Yes with reservations |
|  | If appropriate, treatment following physical violence should include non-occupational post-exposure prophylaxis (nPEP) for HIV and other STIs, as well as prevention of pregnancy according to national standards. | Disposition or Discharge | 43 | 47 | 67 | 50 | Yes |
| Strang et al 2018 | If gender dysphoric adolescent presents in a state of emergency, the first priority is risk reduction/safety. Hospitalization may be necessary in extreme cases to prevent self-harm/mutilation, consult to TGD-competent centre if needed. | Disposition or Discharge | 48 | 31 | 50 | 41 | Yes with reservations |
| Tan et al 2017 | Medications for nPEP should be readily available to TGD persons in ED’s where they are likely to be needed urgently. | Treatment | 81 | 40 | 86 | 64 | Yes |
| Zuniga et al 2015 | Immediate referral of a TGD person to HIV care is recommended following an HIV-positive diagnosis to improve linkage to anti-retroviral therapy. | Disposition or Discharge | 83 | 35 | 81 | 61 | Yes |

Table 3: Interclass Correlation Coefficient by Domain AGREE-II

| Agree II Domain | Interclass Correlation Coefficient | 95% Confidence Interval | Rating |
| --- | --- | --- | --- |
| 1: Scope and purpose | 0.79 | 0.33 to 0.96 | Good |
| 2: Stakeholder involvement | 0.58 | -0.35 to 0.92 | Moderate |
| 3: Rigor of development | 0.87 | 0.59 to 0.98 | Good |
| 4: Clarity of presentation | 0.40 | -0.92 to 0.89 | Poor |
| 5: Applicability | 0.71 | 0.68 to 0.94 | Good |
| 6: Editorial independence | 0.87 | -0.01 to 0.94 | Good |

Table 4: Interclass Correlation Coefficient by Domain, AGREE-REX

| Agree-REX Domain | Interclass Correlation Coefficient | 95% Confidence Interval | Rating |
| --- | --- | --- | --- |
| 1: Clinical Applicability | 0.90 | 0.71 to 0.97 | Good |
| 2: Values and Preferences | -0.067 | -2.1 to 0.71 | Poor |
| 3: Implementability | 0.46 | 0.59 to 0.85 | Poor |
| 4: Total | 0.53 | -0.37 to 0.87 | Moderate |
